# The Angiogenic Effect of microRNA-21 Targeting TIMP3 through the Regulation of MMP2 and MMP9

**DOI:** 10.1371/journal.pone.0149537

**Published:** 2016-02-12

**Authors:** Jianzhong Hu, Shuangfei Ni, Yong Cao, Tao Zhang, Tianding Wu, Xianzhen Yin, Ye Lang, Hongbin Lu

**Affiliations:** 1 Department of Spine Surgery, Xiangya Hospital, Central South University, Changsha, 410008, PR China; 2 Center for Drug Delivery System, Shanghai Institute of Materia Medica, Chinese Academy of Sciences, Shanghai, 201203, PR China; 3 Department of Sports Medicine, Research Centre of Sports Medicine, Xiangya Hospital, Central South University, Changsha, 410008, PR China; Mayo Clinic Minnesota, UNITED STATES

## Abstract

microRNAs are a novel set of small, non-protein-coding nucleotide RNAs that negatively regulate the expression of target mRNAs. miRNA-21 is a microRNA that is highly enriched in endothelial cells. miRNA-21 has been shown to be a potential pro-angiogenic factor in some biological systems. Our previous study showed that the expression of miRNA-21 was up-regulated after spinal cord injury. However, the effect of miRNA-21 on angiogenesis in the spinal cord was unclear. In this study, to understand the role of miRNA-21 on injured endothelial cells exclusively, an oxygen and glucose deprivation model of endothelial cells was constructed, and the up-regulation of miRNA-21 was discovered in this model. An increased level of miRNA-21 by mimics promoted the survival, migration and tube formation of endothelial cells, which simultaneously inhibited tissue inhibitor of metalloproteinase-3 (TIMP3) expression and promoted matrix metalloproteinase-2 (MMP2) and matrix metalloproteinase-9 (MMP9) expression and secretion. A decreased level of miRNA-21 by antagomir exerted an opposite effect. As is well known, survival, migration and tube formation of endothelial cells are necessary prerequisites for angiogenesis after injury. TIMP3 was validated as a direct target of miRNA-21 by dual-luciferase reporter assay. Silencing with small interfering RNA against TIMP3 promoted tube formation and increased MMP2 and MMP9 expression at the protein level. *In vivo*, we found that decreased levels of miRNA-21 inhibited angiogenesis after spinal cord injury in rats using synchrotron radiation micro-computed tomography. In summary, these findings suggest that miRNA-21 has a protective effect on angiogenesis by reducing cell death and promoting cell survival, migration and tube formation via partially targeting the TIMP3 by potentially regulating MMP2 and MMP9. TIMP3 is a functional target gene. Identifying the role of miRNA-21 in the protection of angiogenesis might offer a novel therapeutic target for secondary spinal cord injury, in which angiogenesis is indispensable.

## Introduction

Spinal cord injury (SCI) is a devastating and common central nervous system pathological event that is often accompanied by paralysis and loss of movement or sensation. Epidemiological data show that SCI primarily affects adults whose average age is approximately 41.0 years in the United States[1]. The annual incidence of SCI is approximately 40 cases per million or 12,000 new cases each year[1]. The mechanisms of SCI involve a primary mechanical injury and a secondary injury[2]. The loss of vessel network integrity, ischemia/hypoxia and reperfusion and a significant decrease in microvascular endothelial cells all contribute to secondary injury following SCI[3, 4]. A subsequent neurovascular repair process accompanies SCI[5, 6], which is crucial for functional rehabilitation.

microRNAs (miRNAs) are a novel set of small, non-protein-coding nucleotide RNAs (approximately 19–22 nt) that negatively regulate the expression of target mRNAs at the post-transcriptional level by inhibiting translation or destabilizing target mRNA[7]. Approximately 1000 miRNAs have been found to play critical roles in various patho-physiologic processes in the human genome. To date, several miRNAs have been detected in the mammalian brain and spinal cord, and they seem to play vital roles in some neurological functions[8–10]. miRNA-21 (miR-21) is one of the most widely studied miRNAs because it is overexpressed in almost all human tumors and is considered a oncogene that is involved in apoptosis[11], necrosis[12], invasion[13], proliferation[14] and pro-angiogenesis[15]. Previously, we have detected that miR-21 was one of the most significantly upregulated miRNAs after SCI in a rat model using miRNA microarray; knockdown of miR-21 by antagomir correlated with apoptotic cells and a functional deficit[16]. However, the effect of miR-21 on angiogenesis after SCI remains poorly understood and requires further study.

Angiogenesis, by which new capillaries are formed by sprouting from pre-existing vessels, is a complex process. Postnatal angiogenesis occurs in response to physiological and pathological events, such as reproduction, inflammation, tissue regeneration and tumor growth[17] as well as SCI[18]. In this process, the survival, migration and tube formation of endothelial cells are key factors in the induction of angiogenesis. A recent study has indicated that miR-21 is highly expressed in endothelial cells[19], which suggests that miR-21 may contribute significantly to the functional regulation of endothelial cells. Angiogenesis begins with the degradation of the extracellular matrix (ECM)[20], which is mediated by proteins of the matrix metalloproteinase (MMP) family. Therefore, MMPs catalytically trigger the migration of endothelial cells and the development of neovasculature during angiogenesis[21]. Among MMPs, gelatinases (MMP2 and MMP9) play a leading role in cleaving the ECM[22]. The tissue inhibitor of metalloproteinase-3 (TIMP3) is a member of the TIMP family of proteins, which are expressed ubiquitously as endogenous inhibitors of MMPs[23]. Due to this potent feature, TIMP3 adversely affects persistent endothelial cells migration, thus inhibiting angiogenic potential[24]. In addition, TIMP3 has been shown to induce endothelial cells apoptosis via MMP inhibition[25]. Silencing of TIMP3 promotes prostate cell survival[26]. Previously, we found that TIMP3 may be a direct downstream target gene of miR-21 by bioinformatics prediction (Targetscan).

In the present study, we detected miR-21 expression in human umbilical vein endothelial cell lines (HUVEC), which were deprived of oxygen and glucose (OGD) simulating ischemia-reperfusion injury *in vitro*. To investigate the biologic function of miR-21 in HUVECs of OGD, the expression of miR-21 was artificially up-regulated via mimics or down-regulated via antagomir. Furthermore, we explored the potential mechanisms by examining the effect of miR-21 on TIMP3, MMP2 and MMP9. Our results indicated that miR-21 seems to be able to promote the survival, migration and tube formation of HUVEC of ODG by targeting TIMP3 through MMP2 and MMP9, thus leading to a pro-angiogenic phenotype.

Meanwhile, the synchrotron radiation micro-computed tomography (SRμCT) imaging technique was introduced to achieve high-resolution, non-invasive 3D imaging of the complicated neurovascular networks in the rat spinal cord after injury. The synchrotron radiation light source, which is characterized by high flux density, high coherence, and monochromaticity, could obtain high-resolution imaging of the microvasculature to the submicro-level in the brain, hepatic sinusoids and rabbit eye[27–29]. Using this technique, the effect of miR-21 on the repair process of the microvascular network can be visualized in three dimensions (3D) after SCI in rats.

## Materials and Methods

### Cell culture and transfection

HUVEC lines provided by the Cancer Research Institute of Central South University were purchased from the China Cell Culture Center (Shanghai, China) [30]. Cells were cultured in Roswell Park Memorial Institute (RPMI) 1640 (Gibco) supplemented with 10% fetal bovine serum (FBS) and 100 U/ml penicillin at 37°C with 5% CO_2_ in a humidified atmosphere. Cells were seeded in a 6-well plate at 1×10^5^ cells per well and incubated overnight. Transfection of the miR-21 mimics (50 nM), the miR-21 mimic negative control (50 nM), antagomir-21 (200 nM), antagomir-21 negative control (200 nM), anti-TIMP3 small interfering RNA (siRNA) or anti-TIMP3 siRNA negative control (50 nM) (Ribo, Guangzhou, China) was performed using riboFECT™ CP Transfection Kit (Ribo, Guangzhou, China) according to the manufacturer’s protocol.

### Oxygen glucose deprivation (OGD)

After transfection for 36 h, the cells were subjected to OGD. The culture medium was replaced with glucose-free DMEM (Gibco), and cells were placed into an anaerobic and humidified incubator suffused with 94% N_2_, 1% O_2_ and 5% CO_2_ for 4 h at 37°C[31, 32]. Subsequently, cells were washed twice with RPMI 1640, recovered in their normal culture medium and returned to normoxic incubation conditions for 12–48 h to conduct further experiments.

### Cell survival assay

Cell viability was assessed using a Cell Counting Kit-8 (Dojindo, Kumamoto, Japan) according to the manufacturer’s instructions. Briefly, after transfection and OGD, HUVECs were trypsinized and plated in 96-well plates at 1×10^4^ cells/well. After recovery for 12 h, 24 h or 48h, 10% CCK-8 was added to each well for color conversion. Four hours later, the spectrophotometric absorbance of the purple formazan crystals was measured using a microplate reader at an absorbance wavelength of 450 nm. All of the CCK-8 results were normalized and expressed by subtracting the average optical density reading of the respective cell-free control group. The results were obtained from three independent experiments.

### Hoechst/PI staining

To evaluate cell death after transfection and OGD, HUVECs were stained with Hoechst 33342 and propidium iodide (PI) double fluorescent staining following recovery for 24 h. Briefly, the cells were stained with10 μl of Hoechst 33342 (10 μg /ml, Sigma) in 1 ml basal medium and incubated for 15 min. After washing twice with phosphate-buffered saline **(**PBS), the cells were stained further with 5 μl PI (10 μg/ml, Sigma, USA) in 1 ml buffer solution and incubated for 15 min. Stained cells were fixed by 4% paraformaldehyde and observed under a fluorescent microscope (Olympus BX51, Japan). To quantitatively analyze cell death, the number of cells stained with PI was calculated in 5 random fields. Experiments were performed in triplicate.

### Transwell chamber migration assay

Cell migration was measured using a transwell chamber with 8 μm filter inserts (Corning, Beijing, China) without Matrigel. Cells subjected to transfection and OGD were trypsinized, and a cell suspension was prepared. Approximately 100 μl of cell suspension was added to the upper chamber containing RPMI-1640 medium and 1% BSA at a density of 2×10^5^ cells/ml. The lower chamber was filled with 600 μl RPMI-1640 medium containing 10% FBS. After 24 h, the migrated cells were fixed in 100% methanol for 30 min, and the non-migrated cells were removed. Subsequently, cells on the lower surface of the membrane were stained with 0.1% crystal violet for 20 min and were microscopically observed and counted in 5 random fields. Experiments were performed in triplicate.

### Scratch wound migration assay

HUVECs were inoculated on 6-well plates and incubated overnight. Following transfection and OGD, an approximate confluence rate of 90% was achieved. Subsequently, the cell monolayer was scraped in a cross shape using a 200 μl pipette tip (at time 0). Then, the cells were washed three times with PBS to ensure a scratch area without residual cells and were then recovered for 24 h. Images were captured with a phase-contrast microscope (CKX41; Olympus Corporation, Tokyo, Japan). The distance between the sides of the scratch was measured using the Image-Pro Plus software (Media Cybernetics,Inc., Rockville, MD, USA) in 5 random fields. Experiments were performed in triplicate.

### Capillary network formation assay

The ability of transfected HUVECs subjected to OGD to form capillary networks was evaluated using the In Vitro Angiogenesis Assay Tube Formation Kit (Cultrex®, USA) according to the manufacturer's instructions. Briefly, after transfection and OGD, cells were trypsinized and carefully dispensed in 96-well plates pre-coated with 50 μl Matrigel per well at 1×10^4^ cells/well. Cells were recovered and allowed to form networks for 12 h. To visualize vessels under a fluorescence microscope, the cells were stained with calcein-AM (2 mM) for 15 min, after which pictures were taken using a fluorescence microscope (Olympus BX51, Japan). Quantitative analysis of the network structure was performed using the Image-Pro Plus software (Media Cybernetics, Inc., Rockville, MD, USA) by counting the number of branch nodes in 5 random fields. Experiments were performed in triplicate.

### Real-time RT-PCR

Total RNA was extracted using TRIzol (Invitrogen) according to the manufacturer’s protocol. cDNA was prepared from total RNA using the PrimeScript RT reagent Kit (TaKaRa), and qRT-PCR was performed using the SYBR Premix Ex Taq (TaKaRa) following the specifications. For the quantitative detection of mature miRNA expression, microRNA-specific primers were designed and supplied by RiboBio. miR-U6 was used as an internal control to normalize the relative miR-21 levels. For the quantification of mRNA expression, Primers were provided by Sangon Biotech (Shanghai, China). The expression of GAPDH was used as an internal control. The analysis of gene expression was performed using the 2^-△△Ct^ method. The following primers were used:

miR-21: Forward: 5′-CGGCTAGCTTATCAGACTGA-3′;

            Reverse: 5′-GCAGGGTCCGAGGTATTC-3′.

U6: Forward: 5′-CTCGCTTCGGCAGCACA-3′;

            Reverse: 5′- AACGCTTCACGAATTTGCGT -3′.

TIMP3: Forward: 5′-TTCCACCAAGCACAGTCAAG-3′;

            Reverse: 5′-GACCCAAACCAGAACCAACT-3′.

MMP2: Forward: 5′-AGTTTCCATTCCGCTTCCAG-3′;

            Reverse: 5′-CGGTCGTAGTCCTCAGTGGT-3′.

MMP9: Forward: 5′-CCAACTACGACACCGACGAC-3′;

            Reverse: 5′-TGGAAGATGAATGGAAACTGG-3′.

GAPDH: Forward: 5′-TGGGCTACACTGAGCACCAG-3′;

            Reverse: 5′-AAGTGGTCGTTGAGGGCAAT-3′.

### Western blot

The expression levels of TIMP3, latent forms of MMP2 and MMP9 were measured by western blot analysis. The HUVECs harvested for western blot analysis were lysed by RIPA lysis buffer (50 mM Tris (pH 7.4), 150 mM NaCl, 1% Triton X-100, 1% sodium deoxycholate, 0.1% SDS, 1 mM sodium orthovanadate, 2 mM sodium fluoride, 1 mM EDTA, 0.5 μg/ml leupeptin, 0.7 μg/ml Pepstatin A, 50 μg/ml PMSF, 2.2 μg/ml Aprotinin), and the protein concentrations were determined using a BCA protein assay kit (Pierce). The protocol was performed as described previously[16]. The primary antibodies used in this study were as follows: rabbit-anti-TIMP3 antibody (1:1000, Abcam), mouse-anti-MMP2 antibody (1:1000, Abcam), rabbit-anti-MMP9 antibody (1:1000, Abcam) and anti-GAPDH antibody (1:1000, Abcam) as an internal control.

### Enzyme-linked immunosorbent assay (ELISA)

To detect the secretion of MMP2 and MMP9, the supernatants were collected after transfection and OGD, then assayed by the ELISA kits as described by the manufacturer (Boster, China). The assay was quantified with a spectrophotometric plate reader set at wavelength of 450 nm. Experiments were performed in triplicate.

### Dual-luciferase reporter assay

A luciferase reporter assay with TIMP3 3’UTR (wild seed sequence: ATAAGCTA; mutant seed sequence: CATCTAGC) was used for the analysis (GeneChem Co. Ltd, Shanghai). 293T cells were transfected with TIMP3-3'UTR wild (3'UTR WT), TIMP3-3'UTR mutant (3'UTR MU), TIMP3-3'UTR negative control (3'UTR NC) miR-21 and miR-21 negative control (miR21-NC) in 24-well plates via Lipofectamine 2000 (Invitrogen) according to the manufacturer’s instructions. After 48 h of transfection, firefly and renilla luciferase activities were analyzed with the Luciferase Reporter Assay (Promega) according to the manufacturer’s protocol.

### Experimental animals

All experimental procedures conformed to the Chinese National Health and were approved by the Ethics Committee of the Center for Scientific Research with Animal Models of Central South University (Permit Number: 201303243). The animals were housed individually under specific pathogen free conditions, in identical environments (temperature 22–24°C; humidity 60–80%) on a 12-hour light/dark cycle and fed standard rodent chow ad libitum with free unlimited food and water. A total of 72 adult male Sprague-Dawley rats (250-300g) were randomly assigned to one of two groups: (1) antagomir-21 group (n = 36) and (2) antagomir negative control group (n = 36). Each group was then sub-divided into three time-period sub-groups: 3, 7 and 14 days (n = 12 per sub-group).

### Establishment of contusion SCI model and treatment

All of the rats were anesthetized using an intraperitoneal injection (i.p.) of 10% chloral hydrate at 3 ml/kg (Kermel, Tianjing, China). After laminectomy at thoracic vertebra level 10 (T10) and exposure of the dorsal cord surface with intact dura, moderate contusion injury was induced by a modified Allen^’^s weight drop apparatus (8 g weight at a vertical height of 40 mm, 8 g×40 mm). The emergence of the following phenomena, such as an immediate intramedullary hemorrhage, tail swinging pendulously and both hind limbs retracting convulsively, indicates that the SCI rat model was successfully established. In the antagomir negative control group, all of the SCI rats were treated intrathecally with antagomir negative control (200 nmol/ml, 1μl/h) for 3, 7 or 14 days, using osmotic mini-pumps (Alzet, CA, USA) that were implanted subcutaneously. In the antagomir-21 group, all of the SCI rats were treated intrathecally with antagomir-21 (200 nmol/ml, 1 μl/h) for 3, 7 or 14 days using the same device. After surgery, the rats were housed individually in cages at a constant temperature with free access to food and water and monitored twice a day. A pain relief drug (Tramadol, Grunenthal GmbH, Aachen, Germany) and antibiotic (penicillin sodium, North China pharmaceutical Co, Shijiazhuang, China) were administered once a day within the first three days after surgery. Bladders were manually expressed twice daily until full voluntary or autonomic voiding was obtained. and all efforts were made to minimize suffering. All the animals were healthy and there was no unintended death of animals throughout this study.

### Sample preparation and 3D microvascular imaging using SRμCT

The preparation of experimental samples, SRμCT scanning and 3D rendering were performed as described previously[33]. Briefly, rats in both groups were euthanized (overdose of 10% chloral hydrate, 6 ml/kg, i.p.) and sacrificed at a specific time. A thoracotomy was rapidly performed to expose the heart. Then, vascular system was perfused with heparinized saline at a particular height (110 cm H_2_O) and rate (20 ml/min) via the ascending aorta to allow rapid and sufficient draining of blood flow. After vessel network fixation with 4% paraformaldehyde, the component contrast agents (Microfil MV-122, Flow Tech, CA) were constantly infused into the circulatory system. Subsequently, the spinal cords were carefully dissected out, and 5-mm segments containing the injury site were post-fixed in 4% paraformaldehyde for 24 h at 4°C. All the specimens were dehydrated using a series of graded ethanol (rinsing in 70, 80, 95 and twice in 100% for 4 hours each) followed by SR-μCT scanning at the BL13W1 biomedical beamline in the Shanghai Synchrotron Radiation Facility (SSRF) in China. A total of 720 initial projection images were captured by the CCD detector (Photonic Science, UK), the effective pixel size of which is 3.7×3.7 μm. Subsequently, all of the projected images were transformed into digital slice sections using PITRE software (programmed by the BL13W1 experimental station) based on the filtered back projection (FBP) algorithm. Then, a series of 2D transverse slices were reconstructed into a 3D presentation using the VG Studio Max reconstruction software (Version 2.1, Volume Graphics GmbH, Germany).

### Statistical analysis

Statistical analysis was performed using the SPSS 15.0 software (SPSS Inc, Chicago, IL, USA). Comparisons between different groups were analyzed by one-way ANOVA with a Bonferroni post hoc test. All data were presented as the means±SD, and P <0.05 was considered statistically significant.

## Results

### Regulation of miR-21 expression in HUVECs

**Fig 1:**
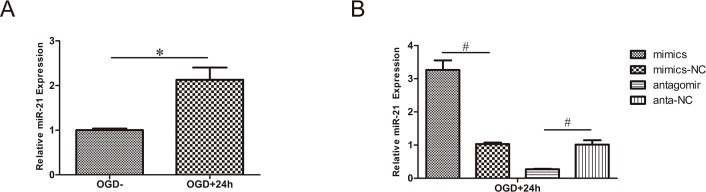
Regulation of miR-21 expression in HUVECs. (A) miR-21 expression of HUVECs exposed to 4 h OGD and recovered for 24 h increased significantly compared with cells without OGD. (B) Evaluation of transfection efficiency by qRT-PCR. After exposure to 4 h OGD and recovery for 24 h, miR-21 mimics increased the expression of miR-21 compared with miR-21 mimic negative control; antagomir-21 decreased the expression of miR-21 compared with antagomir-21 negative control. Values represent means±S.D; n = 3; ^#^p<0.01.

As mentioned above, in our previous study, the level of miR-21 increased after SCI in rats[16]. In the present study, the level of miR-21 increased in HUVECs after treatment for 4 h with OGD and recovery for 24 h (Fig 1A, n = 3/group, p<0.01). This result suggests that miR-21 in the OGD model of HUVECs has a potential protective effect.

qRT-PCR was applied to detect the expression of miR-21 in cells subjected to transfection and OGD. The results revealed that miR-21 expression was significantly higher (3.27±0.40-fold) in the miR-21 mimic group than it was in miR-21 mimic negative control group and that miR-21 expression was significantly lower (0.28±0.01-fold) in the antagomir-21 group than it was in the antagomir-21 negative control group (Fig 1B, n = 3/group, p<0.01).

### Effect of miR-21 on HUVEC death and viability

**Fig 2:**
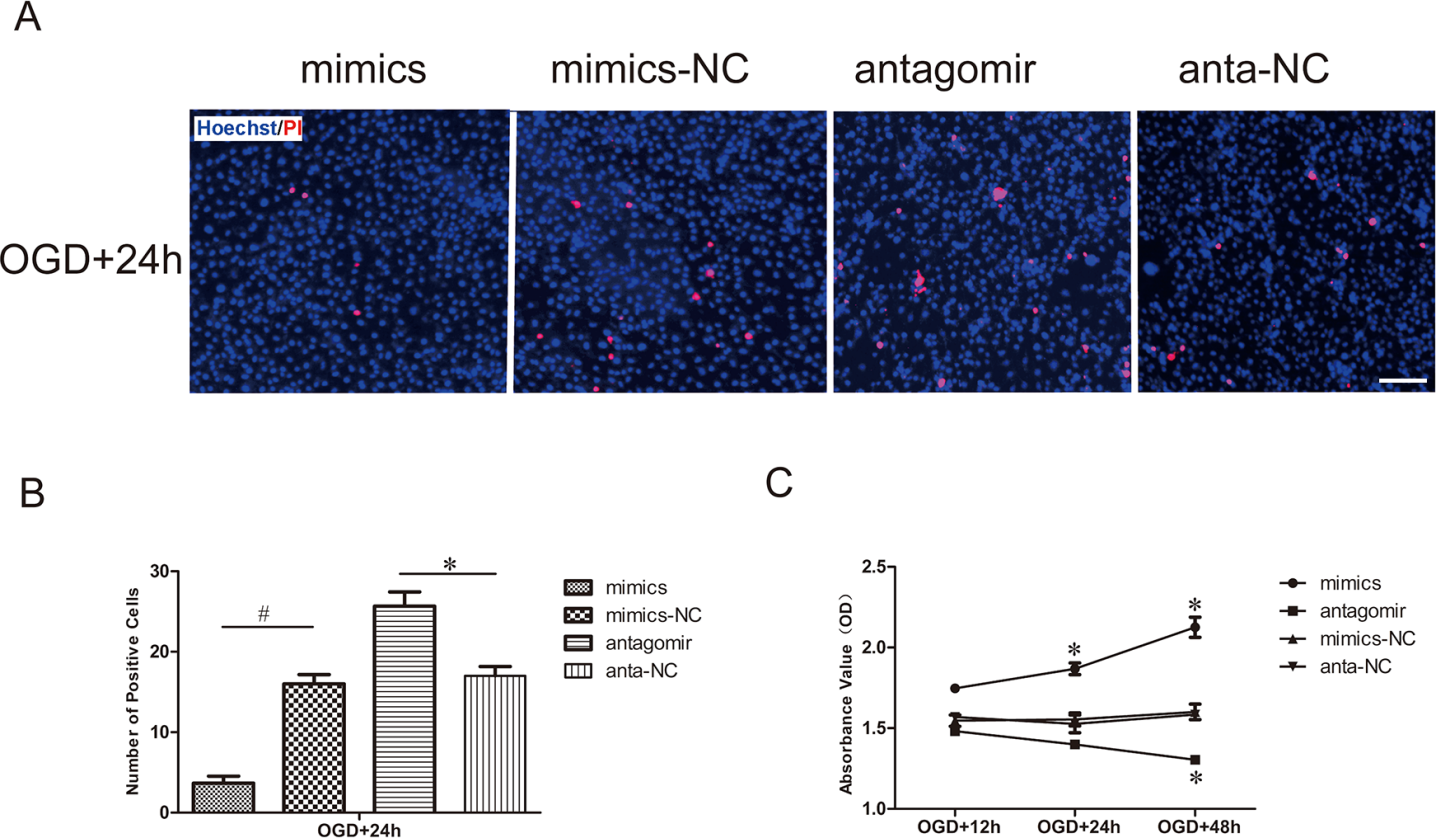
Effect of miR-21 on HUVEC death and viability. (A) Detection of HUVEC death transfected with miR-21 mimics, miR-21 mimic negative control, antagomir-21 or antagomir-21 negative control and then exposed to OGD by Hoechst/PI staining. The cell death was evaluated by double fluorescent staining with Hoechst (blue) and PI (red). Bar = 100 μm. (B) The statistical data show changes in the numbers of dead cells in different groups. Values represent means±S.D; n = 3; *p<0.05, ^#^p<0.01. (C) The viability of HUVECs was detected by CCK-8 assay. Comparision of viability index between the treatment groups and respective negative groups was assessed at 12 h, 24 h and 48 h of recovery after transfection and OGD. Values represent means±S.D; n = 3; *p<0.05.

Hoechst is a reactive dye that combines with DNA specifically and is capable of entering the normal cytomembrane and staining the nucleolus blue. In contrast to Hoechst, PI is a nucleic acid dye that only has access to impaired cytomembrane and stains the nucleolus red. Hoechst/PI dual-staining in lavender represents cell death. In our results, the number of dead cells was far less in the miR-21 mimic group than it was in the miR-21 mimic negative control group after the aforementioned OGD and recovery. In contrast, the number of dead cells was far greater in the antagomir-21 group than it was in the antagomir-21 negative control group (Fig 2A). Statistical data revealed that miR-21 mimics significantly decreased cell death (0.23±0.09-fold) compared with the miR-21 mimic negative control and that antagomir-21 significantly increased cell death (1.51±0.18-fold) compared with the antagomir-21 negative control (Fig 2B, n = 3/group, p<0.05).

In addition, we explored the potential impact of miR-21 on HUVEC viability via a CCK-8 assay. Our results indicated that the cell viability was expedited in miR-21 mimic-transfected HUVECs compared with miR-21 mimic negative control-transfected HUVECs after OGD and recovery for 24 h and 48 h; conversely, antagomir-21 could significantly inhibit the viability of HUVECs compared with antagomir-21 negative control-transfected HUVECs after OGD and recovery for 48 h (Fig 2C, n = 3/group, p<0.05).

### Effect of miR-21 on HUVEC migration

**Fig 3:**
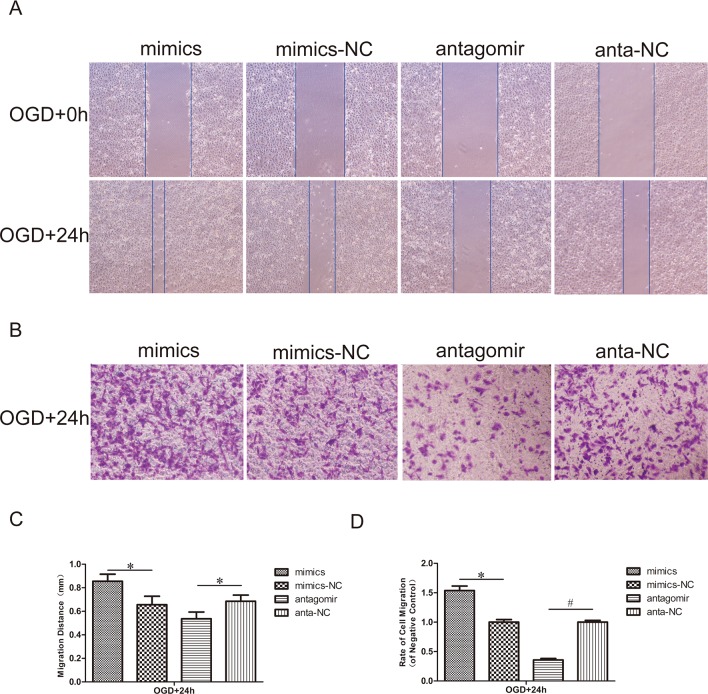
Effect of miR-21 on HUVEC migration. (A) Scratch wound migration assay *in vitro*. After transfection with miR-21 mimics, miR-21 mimic negative control, antagomir-21 or antagomir-21 negative control, confluent HUVECs monolayers were exposed to OGD and then scratched to induce horizontal migration of HUVECs (magnification, x100). (C) Comparison of the distance of HUVECs horizontal migration at 0 h and 24 h between treatment groups and negative control groups. Values represent means±S.D; n = 3; *p<0.05. (B) Transwell chamber migration assay *in vitro*. HUVECs subjected to transfection and OGD mentioned above were trypsinized and seeded into the upper chamber and then allowed to vertically migrate for 24 h. Cells on the lower surface of the membrane were stained with 0.1% crystal violet (lavender) (magnification, x100). (D) Comparison of the rate of cell migration in the treatment groups relative to their respective negative control groups at 24 h. Values represent means±S.D; n = 3; *p<0.05, ^#^p<0.05.

To assess the effect of miR-21 on the mobility of HUVECs, a scratch wound migration assay and transwell chamber migration assay were performed. In the scratch wound migration assay (Fig 3A and 3C), the horizontal migration distance of HUVECs that had been transfected with antagomir-21 and exposed to OGD was significantly reduced compared with the antagomir-21 negative control (0.77±0.12-fold, n = 3/group, p<0.05). Inversely, the horizontal migration distance of HUVECs transfected with miR-21 mimics and exposed to OGD was significantly increased compared with the miR-21 mimic negative control (1.30±0.13-fold, n = 3/group, p<0.05). In the transwell chamber migration assay (Fig 3B and 3D), the vertical migration rate of HUVECs transfected with antagomir-21 and exposed to OGD was significantly reduced compared with the antagomir-21 negative control (0.36±0.04-fold, n = 3/group, p<0.01). Inversely, the vertical migration rate of HUVECs transfected with miR-21 mimics and exposed to OGD was significantly increased compared with the miR-21 mimic negative control (1.54±0.11-fold, n = 3/group, p<0.05).

### Effect of miR-21 on the capillary network formation of HUVECs

**Fig 4:**
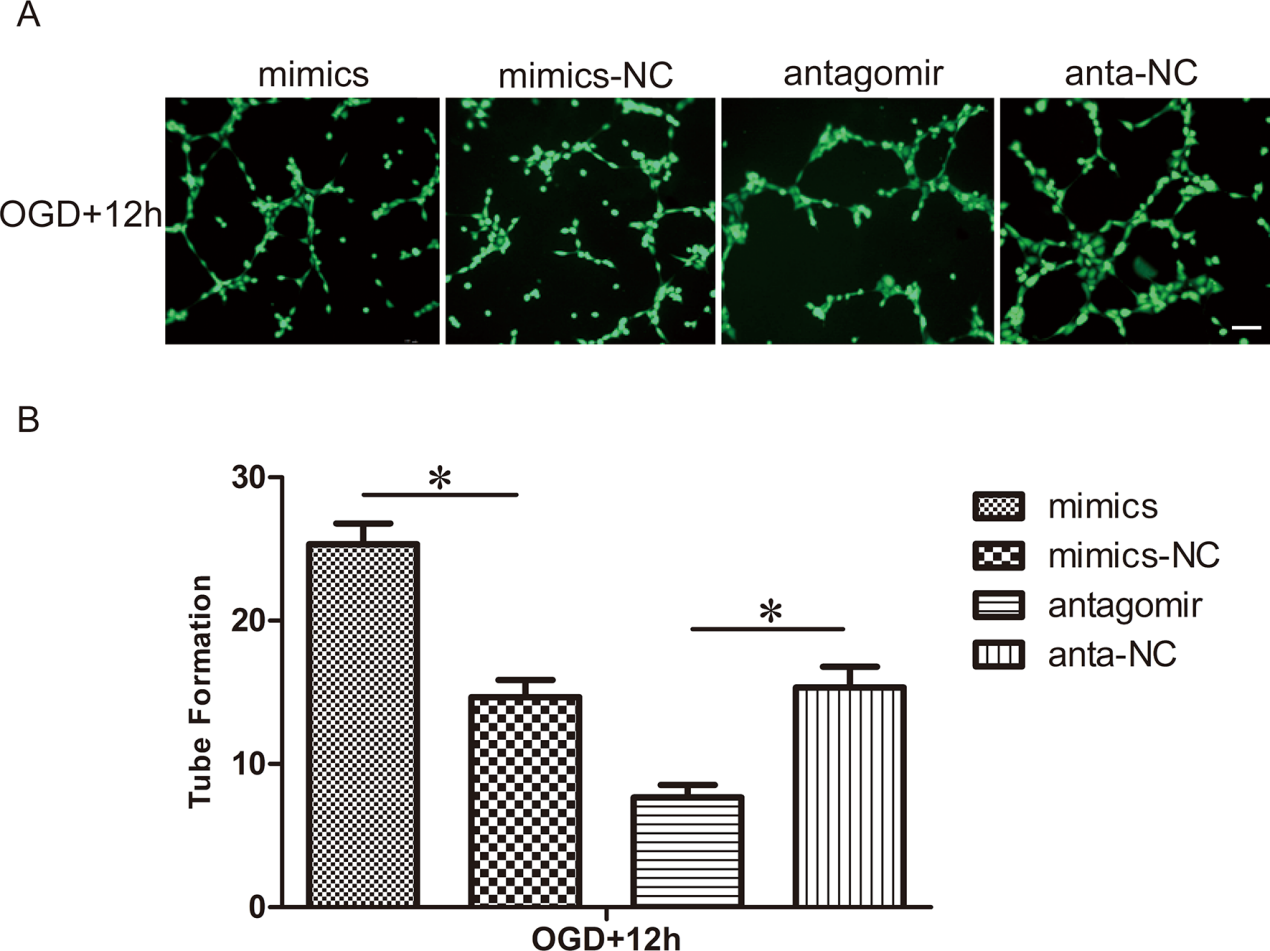
Effect of miR-21 on capillary network formation of HUVECs. (A) After transfection with miR-21 mimics, miR-21 mimic negative control, antagomir-21 or antagomir-21 negative control, HUVECs were exposed to OGD and then trypsinized and seeded onto Matrigel. Cells were incubated and allowed to form networks for 12 h, then stained with calcein-AM (Green) for 15 min. Bar = 200 μm. (B) Comparison of the number of branch nodes in the treatment groups relative to respective negative control groups at 12 h. Values represent means±S.D; n = 3; *p<0.05.

We examined the role of miR-21 in the tube formation of HUVECs using a capillary network formation assay (Fig 4A and 4B). The number of branch nodes of HUVECs transfected with antagomir-21 and exposed to OGD was significantly lower than those transfected with the antagomir-21 negative control (0.50±0.08-fold, n = 3/group, p<0.05). On the contrary, the number of branch nodes of HUVECs transfected with miR-21 mimics and exposed to OGD was clearly higher than those transfected with the miR-21 mimic negative control (1.73±0.14-fold, n = 3/group, p<0.05).

### Effect of miR-21 on TIMP3, MMP2 and MMP9 expression

**Fig 5:**
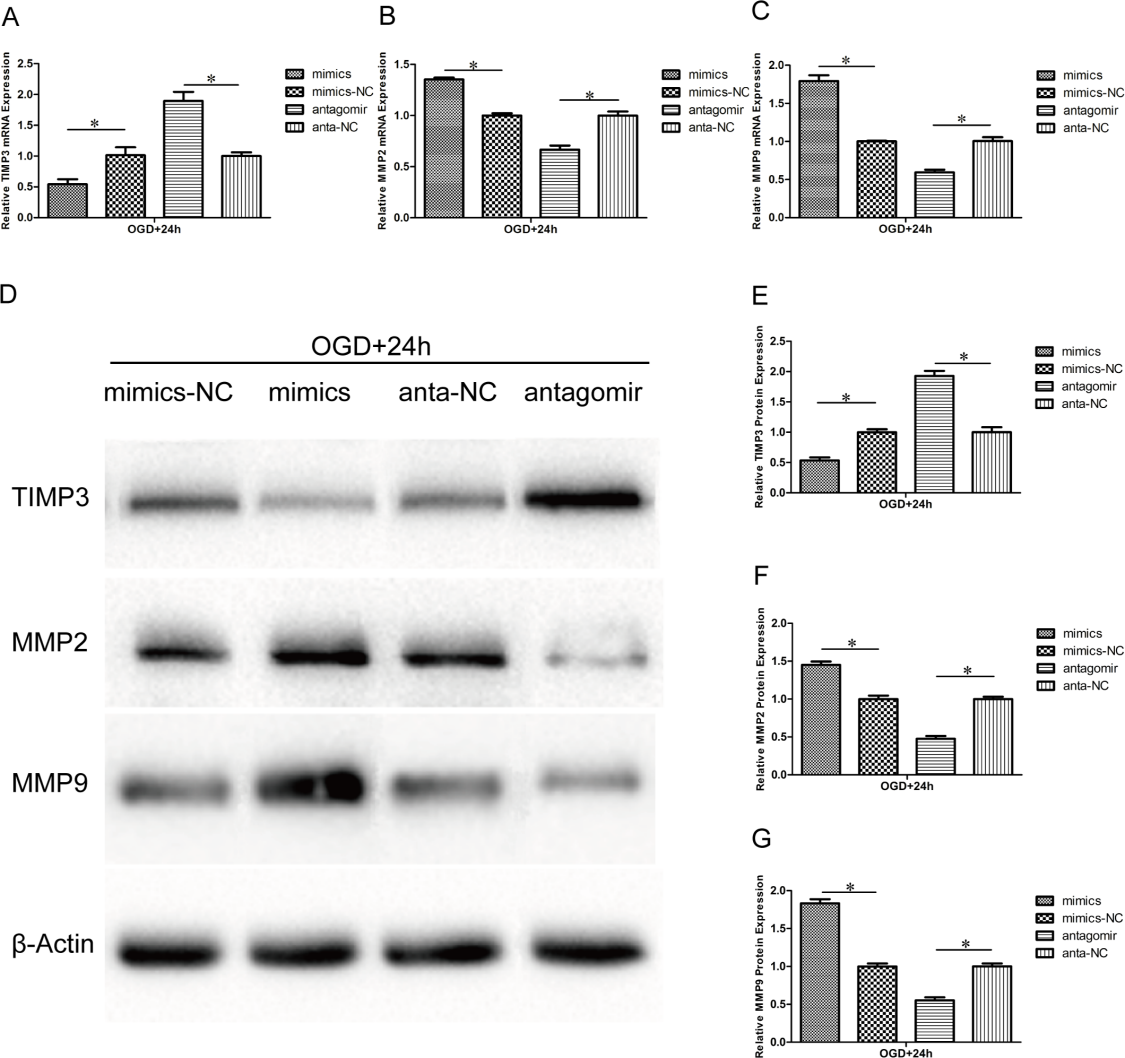
Effect of miR-21 on TIMP3, MMP2 and MMP9 expression. HUVECs were transfected with miR-21 mimics, miR-21 mimic negative control, antagomir-21 or antagomir-21 negative control and exposed to OGD for 4 h and then recovered for 24h. (A-C) qRT-PCR reveals the different mRNA expression levels of TIMP3, MMP2 and MMP9 between the treatment groups and respective negative groups. Values represent means±S.D; n = 3; *p<0.05. (D) Western blot bands show the protein expression of TIMP3, MMP2 and MMP9. (E-G) Comparison of TIMP3, MMP2 and MMP9 protein expression between the treatment groups and respective negative groups. Values represent means±S.D; n = 3; *p<0.05.

Previous reports have shown that miR-21 targeted TIMP3 and decreased its expression in some cancer cells[34, 35]. However, the relation between miR-21 and TIMP3 in HUVECs after OGD is currently unclear. MMP2 and MMP9 can be inhibited by TIMP3 and are involved in both the degradation of the ECM and the migration of endothelial cells. In this study, the expression of TIMP3, MMP2 and MMP9 in HUVECs of the aforementioned transfectsion and OGD was detected by qRT-PCR at the mRNA level and by western blot at the protein level. In the miR-21 mimic group, TIMP3 expression was decreased at both the mRNA (Fig 5A, 054±0.12-fold, n = 3/group, p<0.05) and protein levels (Fig 5D and 5E, 053±0.09-fold, n = 3/group, p<0.05) relative to the miR-21 mimic negative control group, whereas the expression of both MMP2 and MMP9 was increased at both the mRNA (Fig 5B and 5C, 1.35±0.02-fold and 1.79±0.11-fold, respectively, n = 3/group, p<0.05) and protein levels (Fig 5D, 5F and 5G, 1.45±0.08-fold and 1.83±0.09-fold, respectively, n = 3/group, p<0.05) relative to the miR-21 mimic negative control group. In contrast, in the antagomir-21 group, the expression of TIMP3 (Fig 5A, 5D and 5E, n = 3/group, p<0.05), MMP2 (Fig 5B, 5D and 5F, n = 3/group, p<0.05) and MMP9 (Fig 5C, 5D and 5G, n = 3/group, p<0.05) was reversed.

### Effect of miR-21 on MMP2 and MMP9 secretion

**Fig 6:**
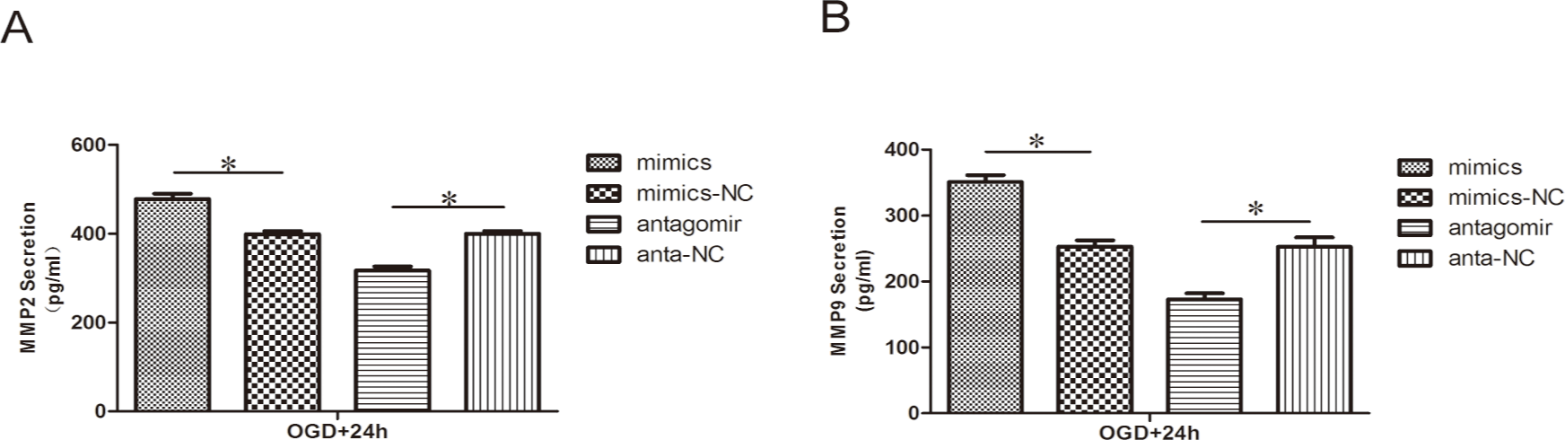
Effect of miR-21 on MMP2 and MMP9 secretion. ELISA reveals the different secretion levels of MMP2 (A) and MMP9 (B) between the treatment groups and respective negative groups. Values represent means±S.D; n = 3; *p<0.05.

To determine whether the secretion of MMP2 and MMP9 was altered by miR-21, the content of these protein in the conditioned media was evaluated by ELISA. Similar to the effects on protein expression, In the miR-21 mimic group, the secretion of both MMP2 (Fig 6A, 1.20±0.04-fold, n = 3/group, p<0.05) and MMP9 (Fig 6B, 1.39±0.06-fold n = 3/group, p<0.05) was increased relative to the miR-21 mimic negative control group. In contrast, in the antagomir-21 group, the secretion of both MMP2 (Fig 6A, 0.79±0.03-fold, n = 3/group, p<0.05) and MMP9 (Fig 6B, 0.68±0.05-fold, n = 3/group, p<0.05) was decreased relative to the antagomir-21 negative control group.

### TIMP3 is a direct target of miR-21

**Fig 7:**
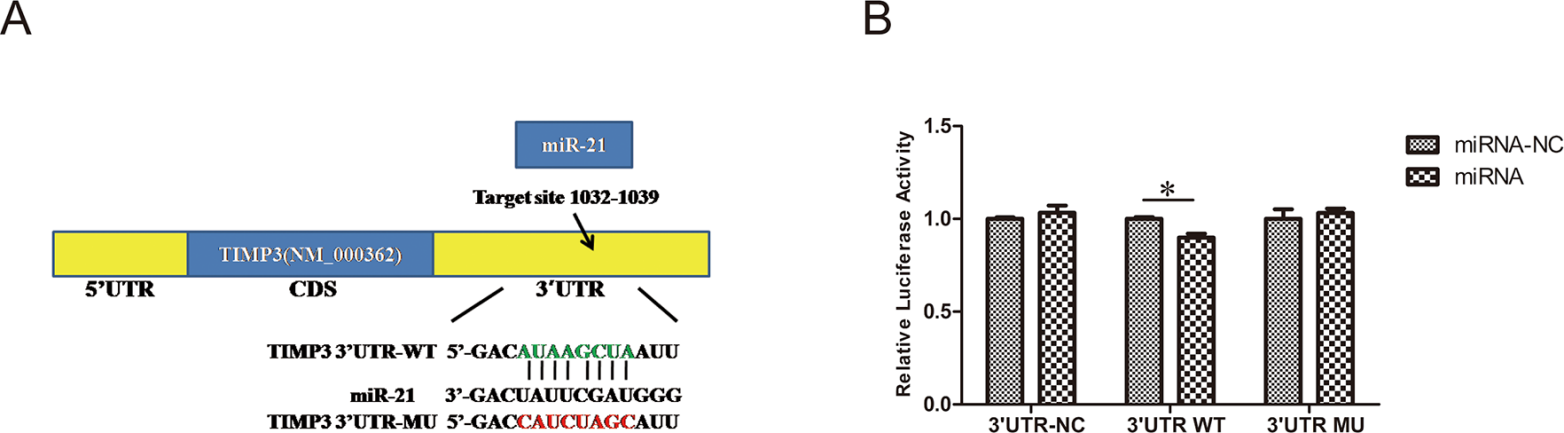
The 3’-UTR of TIMP3 is a target for miR-21. (A) Predicted miR-21 binding sites within the 3’-UTR of TIMP3 mRNA. (B) Validation of the binding sites of miR-21 in TIMP3 by dual-luciferase reporter assay. A fragment of the 3’-UTR TIMP3 mRNA with the putative miR-21 binding sequence was cloned into a firefly luciferase reporter constructed into 293 T cells with miR-21 and miR21-NC. Firefly luciferase activity was normalized to Renilla luciferase expression (n = 3, *P<0.05 compared with miR-21-NC group).

To further confirm that TIMP3 is a direct target gene of miR-21, we cloned a construct with a fragment of the 3’-UTR of TIMP3 mRNA with the putative miR-21 binding sequence into a firefly dual-luciferase reporter vector for co-transfection with vehicle control into 293T cells. When we co-transfected 3’-UTR-WT with miR-21, the luciferase activity was changed to 0.90±0.04-fold compared with cells that had been co-transfected with miR-21-NC (Fig 7, n = 3/group, p<0.05). These data therefore indicate that miR-21 could directly bind to TIMP3.

### Silencing TIMP3 in HUVECs promotes the expression of MMP2 and MMP9 and enhances capillary network formation

**Fig 8:**
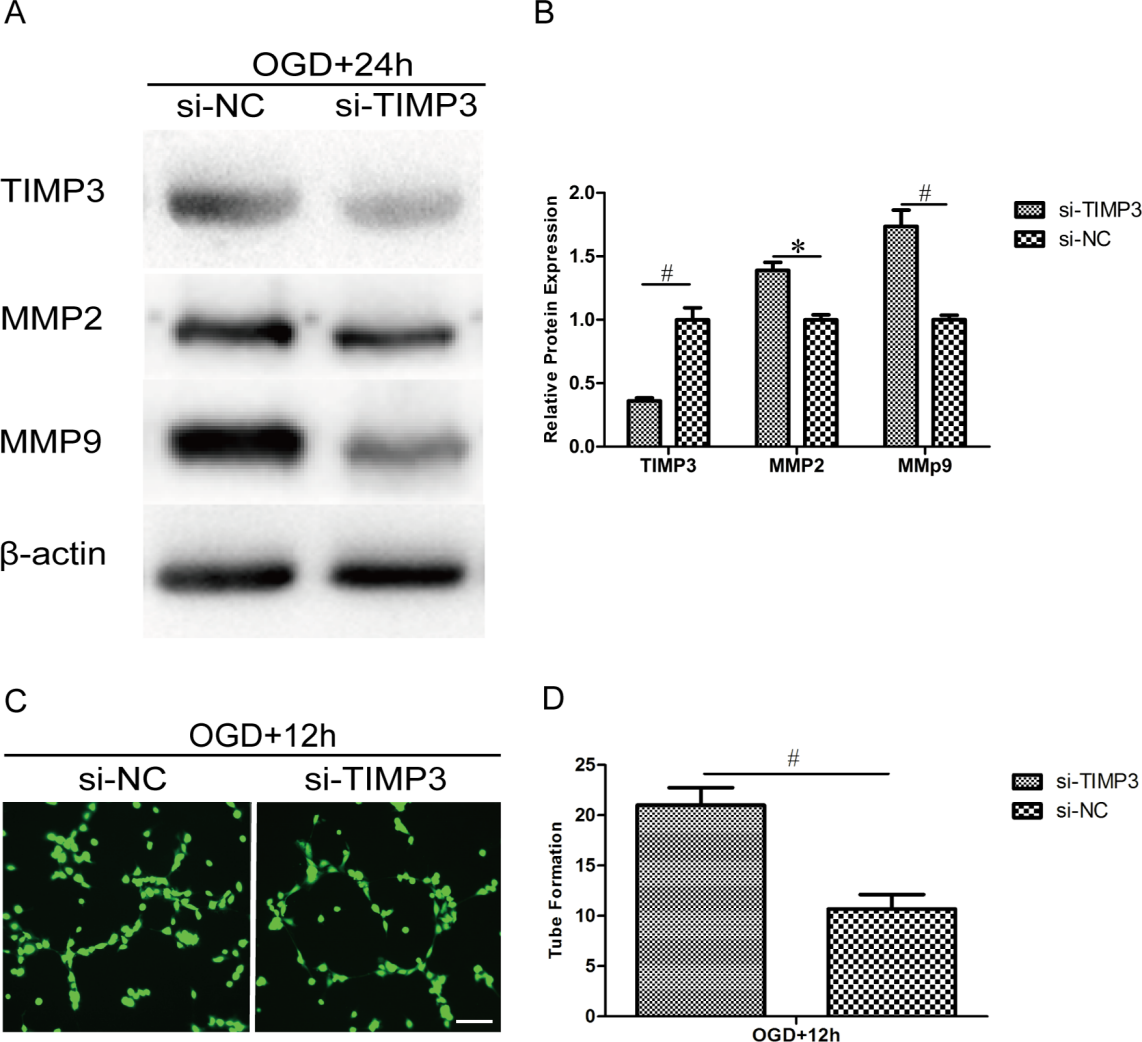
Silencing of TIMP3 in HUVECs promotes the expression of MMP2 and MMP9 and enhances capillary network formation. HUVECs were transfected with siRNA or negative control targeting TIMP3 and exposed to OGD for 4 h and then recovered for 12 h or 24 h. (A) HUVECs were subjected to western blot analysis to confirm the silencing efficiency of TIMP3 siRNA and its effect on the expression of MMP2 and MMP9. (B) A comparison of TIMP3, MMP2 and MMP9 protein expression between the siRNA group and negative control group. Values represent means±S.D; n = 3; *p<0.05, ^#^p<0.01. (C) HUVECs were subject to a capillary network formation assay for 12 h and then stained with calcein-AM (Green) for 15 min. Bar = 200 μm. (D) Comparison of the number of branch nodes in the siRNA group relative to the negative control group. Values represent means±S.D; n = 3; ^#^p<0.01.

The above work has shown that miR-21 may enhance angiogenesis by targeting TIMP3 through the up-regulation of MMP2 and MMP9 expression. To determine whether this effect is altered by the silencing of TIMP3, we delivered siRNA targeting TIMP3 into HUVECs followed by OGD. The silencing efficiency of TIMP3 and its impact on the expression of MMP2 and MMP9 were confirmed by western blot (Fig 8A). Statistical analysis showed that the expression of TIMP3 was decreased significantly by siRNA at the protein level (Fig 8B, 0.36±0.03-fold, n = 3/group, p<0.01) relative to the siRNA negative control and that the expression of MMP2 and MMP9 was increased by siRNA at the protein level (Fig 8B, 1.39±0.09-fold, p<0.05 and 1.74±0.18-fold, p<0.01, respectively, n = 3/group) relative to the siRNA negative control. The capillary network formation assay showed that transfection with TIMP3 siRNA significantly improved tube formation after OGD (Fig 8C). Statistical analysis showed that the number of branch nodes in the TIMP3 siRNA group was significantly higher (Fig 8D, 1.97±0.23-fold, n = 3/group, p<0.01) than that in the TIMP3 siRNA negative control group. These results suggest that TIMP3 is essential for miR-21-enhanced angiogenesis by potentially regulating MMP2 and MMP9.

### 3D vascular morphology changes and quantitative analysis after injury *in vivo*

**Fig 9:**
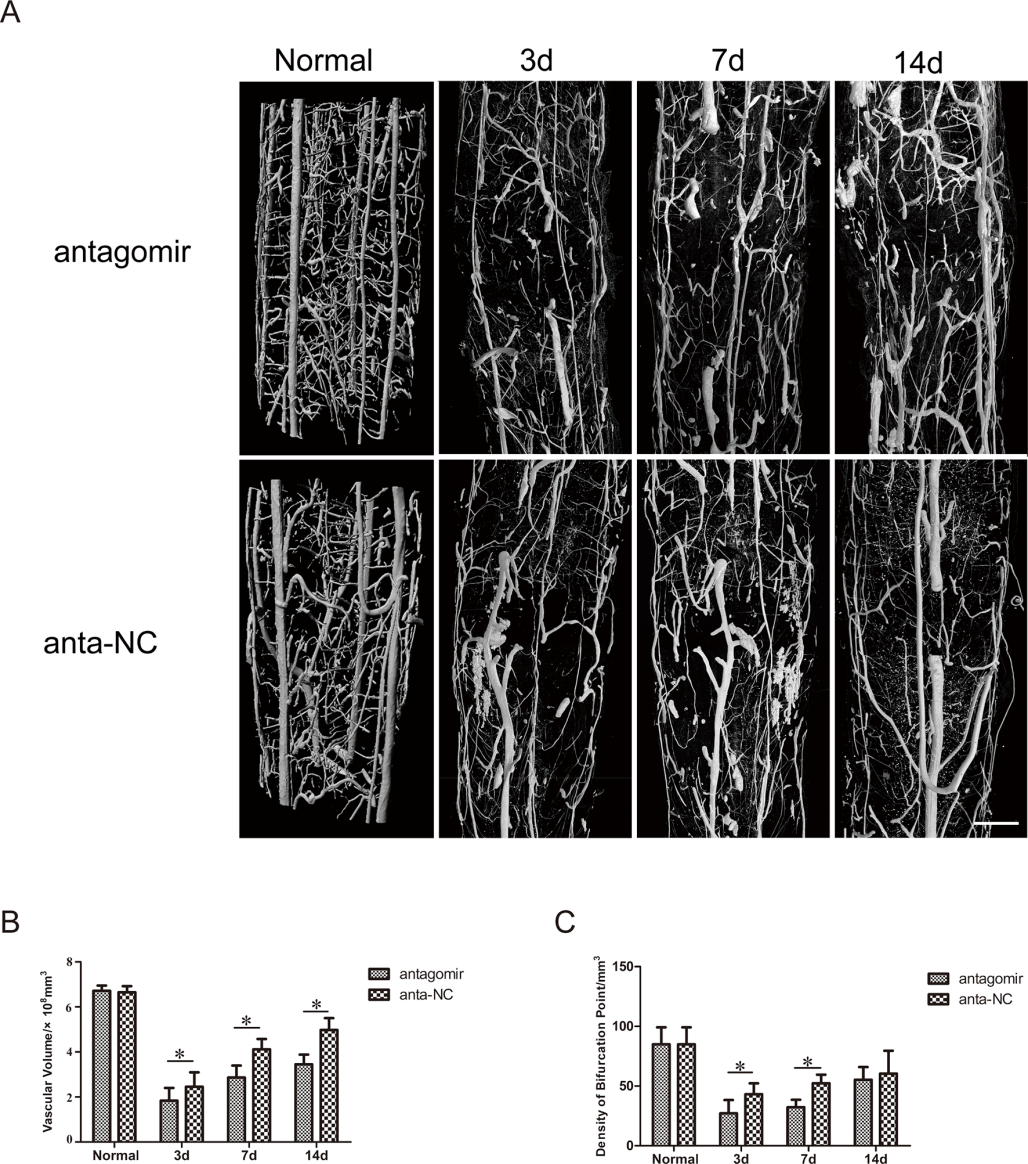
3D vascular morphologic changes and quantitative analysis after injury *in vivo*. (A) 3D vascular morphology changes after SCI in rats treated with antagomir-21 or antagomir-21 negative control. (B) Effect of antagomir-21 on the density of bifurcation points (DBP). (C) Effect of antagomir-21 on the sum of vascular volume (SVV). These results revealed that the down-regulation of miR-21 was adverse to remodeling of the vasculature 3,7 or 14 days after SCI in rats (Bar = 500 μm, Values represent means±S.D; n = 3; *p<0.05).

The microvasculature both in the antogomir-21 group and the antagomir-21 negative control group was severely damaged following SCI. Three days after SCI, the large extramedullary blood vessels, such as the anterior spinal artery and posterior spinal vein, were generally re-perfused preliminarily. In contrast, serious distortion, rupture, displacement and obstruction mainly occurred in intramedullary capillaries and could be visualized in two groups, which inevitably resulted in a severe imbalance in the intramedullary blood supply network and a gross inadequacy in the blood perfusion of white matter and gray matter in damaged sections (T10 level). Damage of the microvasculature occurred in adjacent areas of injury as well. As time passed, repair of the microvasculature of the spinal cord occurred gradually. Seven and fourteen days after SCI, vascular perfusion, vascular continuity and orderliness had significantly recovered and improved compared with those observed three days after SCI. Improvements of continuity and orderliness were more obvious in the antagomir-21 negative control group than they were in the antagomir-21 group (Fig 9A). However, they still could not recover to normal levels, which suggested that vascular remodeling after SCI would be a long process.

For an in-depth understanding of the 3D vessel morphometry associated with injured spinal cord vasculature in both groups, the sum of the vascular volume (SVV, Fig 9B) and the density of bifurcation points (DBP, number of vascular nodes per unit volume, Fig 9C) were calculated, which can partially reflect angiogenesis and re-perfusion of blood flow. Three days after SCI, the SVV and DBP were significantly reduced. With the passage of time, SVV and DBP gradually increased. Through statistical analysis, we found that the recovery of SVV was more prominent in the antagomir-21 negative control group than in the antagomir-21 group at all three time points (P<0.05). Three and seven days after SCI, the DBP increased more significantly in the antagomir-21 negative control group than in the antagomir-21 group (p<0.05), but there was no significant difference in the DBP of either group fourteen days after SCI.

## Discussion

miRNAs can regulate gene expression and are thus involved in a variety of neurobiological processes, including cell growth, differentiation, proliferation and neural activity, as well as SCI induced by multiple pathogenic events, such as inflammation, oxidation, demyelination and apoptosis[8, 36]. Among these miRNAs, miR-21 was overexpressed and found to protect neurons after SCI in rats as described previously[16]. Moreover, the hypertrophic response to SCI in astrocytes was attenuated by the overexpression of miR-21[37]. As is common knowledge, the initial mechanical injury triggers a series of complex secondary processes involving the nerves and blood vessels that largely determine the functional outcome of SCI[38]. Angiogenesis is required for functional repair and metastasis after SCI. In fact, miR-21 is up-regulated in diverse tumors and plays a potential role in the angiogenesis of cancers[15, 39]. In addition, another study demonstrated that knock-down of miR-21 had a distinct negative effect on neointimal lesion formation, thus suggesting a pro-angiogenic effect of miR-21 by possibly regulating PTEN and Bcl-2[40]. miR-21 had been manifested to promote migration and tube formation of uninjured HUVEC in vitro[41]. However, the direct role of miR-21 in the regulation of angiogenesis in response to SCI remains to be revealed.

Our present study confirmed that miR-21 was able to promote angiogenesis after SCI both *in vitro* and *in vivo*. First, to clarify the role of miR-21 on injured endothelial cells exclusively and the specific mechanisms, the OGD model of HUVECs was constructed, and the up-regulation of miR-21 was discovered in this model, which was consistent with the altered miR-21 expression observed in an SCI model of rats[16]. As is well known, the survival, migration and tube formation of endothelial cells are necessary prerequisites for angiogenesis after injury. In the present study, the over-expression of miR-21 by mimics or the down-regulation of miR-21 by antagomir was implemented successfully in HUVECs. miR-21 mimics inhibited cell death and activated cell proliferation, migration and tube formation. Conversely, antagomir-21 promoted cell death and inhibited proliferation, migration and tube formation. These results indicate that miR-21 plays a vital role in modulating angiogenesis *in vitro*. Subsequently, we want to investigate the mechanism and relevant signaling molecules in the miR-21-mediated induction of angiogenesis.

Because of reduced growth and anti-angiogenic activity, TIMP3 is considered to be a tumor suppressor in various tumors[24, 42]. TIMP3, mainly through the inhibition of MMP activity, plays a potent role in inhibiting angiogenesis by modulating ECM remodeling[42]. Another review indicated that degradation of the vascular basement membrane and remodeling of the ECM regulated by MMPs allow endothelial cells to migrate and are necessary for angiogenesis[43]. Among the MMPs, gelatinases (MMP2 and MMP9) play a leading role in cleaving the ECM[22]. An inhibitor of MMP activities is not conductive to tumor metastasis and angiogenesis[44]. Absence of MMP-2 and MMP-9 inhibits experimental choroidal neovascularization[45]. So, it can be speculated that TIMP3 through inhibiting MMPs, might prevent angiogenesis. In addition, the pro-apoptotic function has been ascribed to TIMP3. The pro-apoptosis domain of TIMP3 has been demonstrated to be localized to the N-terminal three loops that associate with functional MMP inhibitory activity[46]. There are many studies, however, that suggest that A-Disintegrin and Metalloproteases family (ADAMs) inhibited by TIMP3 may be responsible for this function of TIMP3[47–49].

In kidney injury, TIMP3 also participates in the stabilization of capillary networks during angiogenesis, which is important for newly forming capillary networks[50]. TIMP3 knockout pericytes invaded more effectively and formed more functional blood vessels *in vitro*.

However, the role of TIMP3 in angiogenesis after CNS injury has been scarcely reported. MMPs participate in the re-growth of damaged vessels, along with the initiation of recovery and angiogenesis after stroke[51].

These studies suggest that TIMP3 may exert an anti-angiogenic effect by inhibiting MMP2 and MMP9. In our study, mimics of miR-21 could inhibit TIMP3 expression and promote MMP2 and MMP9 expression and secretion in an OGD model of HUVECs. In contrast, antagomir-21 could promote TIMP3 expression and inhibit MMP2 and MMP9 expression and secretion. TIMP3 has proven to be a target gene of miR-21 in adual-luciferase reporter assay. Silencing TIMP3 exerted a pro-angiogenic effect *in vitro*. These results imply that miR-21 may be involved in endothelial cell survival, migration and capillary formation by inhibiting TIMP3 through the promotion of MMP2 and MMP9 activity, thus furthering angiogenesis *in vitro*. In addition, TIMP3 also exhibits anti-angiogenic activity by binding directly to VEGF receptor 2 and blocking the action of VEGF on endothelial cells[52] or by binding to angiotensin II type 2 receptor[53], which is independent of its MMP inhibitory activities.

Second, we found that inhibition of miR-21 by antagomir is not conducive to angiogenesis three, seven or fourteen days after SCI using absorption-contrast imaging of SRμCT i*n vivo*. SRμCT, which can detect vessels with diameters as small as approximately 7.4 μm[54], provides new insight into the effect of miR-21 on the vascular reaction to spinal cord trauma in three dimensions. Currently, SRμCT mainly possesses unique ultra-high resolution and non-destructive features and has been gradually applied to microvascular imaging and microscopic analysis of the central nervous system under conditions of physiologic and pathologic stress.

It is important to recognize the limitations of our study. The exact role of MMP2 and 9 in miR-21 mediated angiogenesis is not verified. This is the further research direction. In spite of it, this study indicated that miR-21 could play a protective role in angiogenesis through targeting TIMP3.

## Conclusions

In summary, in an SCI model in rats and an ischemia/reperfusion injury model in HUVECs, miR-21 has a protective effect on angiogenesis. It may be achieved by reducing cell death and promoting cell survival, migration and tube formation by partially targeting TIMP3 via potentially regulating MMP2 and MMP9. Identifying the roles of miR-21 in angiogenic protection might offer a novel therapeutic target for secondary SCI, in which angiogenesis is indispensable.

## Supporting Information

S1 File ARRIVE Guidelines Checklist. (PDF)
